# OrchidBase 5.0: updates of the orchid genome knowledgebase

**DOI:** 10.1186/s12870-022-03955-5

**Published:** 2022-12-02

**Authors:** You-Yi Chen, Chung‐I Li, Yu-Yun Hsiao, Sau-Yee Ho, Zhe-Bin Zhang, Chien-Chi Liao, Bing-Ru Lee, Shao-Ting Lin, Wan-Lin Wu, Jeen-Shing Wang, Diyang Zhang, Ke-Wei Liu, Ding-Kun Liu, Xue-Wei Zhao, Yuan-Yuan Li, Shi-Jie Ke, Zhuang Zhou, Ming-Zhong Huang, Yong-Shu Wu, Dong-Hui Peng, Si-Ren Lan, Hong-Hwa Chen, Zhong-Jian Liu, Wei-Sheng Wu, Wen-Chieh Tsai

**Affiliations:** 1 Institute of Tropical Plant Sciences and Microbiology, National Cheng Kung University, Tainan, 701 Taiwan; 2 Department of Statistics, National Cheng Kung University, Tainan, 701 Taiwan; 3 Orchid Research and Development Center, National Cheng Kung University, Tainan, 701 Taiwan; 4 Department of Electrical Engineering, National Cheng Kung University, Tainan, 701 Taiwan; 5 Key Lab of National Forestry and Grassland Administration for Orchid Conservation and Utilization and International Orchid Research Center at College of Landscape Architecture, Fujian Agriculture and Forestry University, Fuzhou, Fujian 350002 China; 6 Tsinghua-Berkeley Shenzhen Institute (TBSI), Center for Biotechnology and Biomedicine, Shenzhen Key Laboratory of Gene and Antibody Therapy, State Key Laboratory of Chemical Oncogenomics, State Key Laboratory of Health Sciences and Technology, Institute of Biopharmaceutical and Health Engineering (iBHE), Shenzhen International Graduate School, Tsinghua University, Shenzhen, 518055 China; 7 Zhejiang Institute of Subtropical Crops, Zhejiang Academy of Agricultural Sciences, Wenzhou, 325005 China; 8 Education Botanical Garden of Fujian Agriculture and Forestry University, Fuzhou, Fujian 350002 China; 9 Department of Life Sciences, National Cheng Kung University, Tainan, 701 Taiwan; 10 Institute of Vegetable and Flowers, Shandong Academy of Agricultural Sciences, Jinan, 250100 China

**Keywords:** Orchid, OrchidBase, Whole-genome sequences, Orchidoideae, *Platanthera*, Synteny, Gene order, miRNA

## Abstract

Containing the largest number of species, the orchid family provides not only materials for studying plant evolution and environmental adaptation, but economically and culturally important ornamental plants for human society. Previously, we collected genome and transcriptome information of *Dendrobium catenatum*, *Phalaenopsis equestris*, and *Apostasia shenzhenica* which belong to two different subfamilies of Orchidaceae, and developed user-friendly tools to explore the orchid genetic sequences in the OrchidBase 4.0. The OrchidBase 4.0 offers the opportunity for plant science community to compare orchid genomes and transcriptomes and retrieve orchid sequences for further study.

In the year 2022, two whole-genome sequences of Orchidoideae species, *Platanthera zijinensis* and *Platanthera guangdongensis*, were de novo sequenced, assembled and analyzed. In addition, systemic transcriptomes from these two species were also established. Therefore, we included these datasets to develop the new version of OrchidBase 5.0. In addition, three new functions including synteny, gene order, and miRNA information were also developed for orchid genome comparisons and miRNA characterization.

OrchidBase 5.0 extended the genetic information to three orchid subfamilies (including five orchid species) and provided new tools for orchid researchers to analyze orchid genomes and transcriptomes. The online resources can be accessed at https://cosbi.ee.ncku.edu.tw/orchidbase5/

## Background

Orchids have been considered mysterious and fascinating to scientists, collectors, growers, and general public. Orchidaceae represents approximately 10 percent of angiosperms and is one of the largest angiosperm family containing more than 25,000 species categorized in about 900 genera [1]. The basic blueprint of orchid flower including three sepals at outermost floral whorl, two lateral petals and one elaborated medial labellum at second whorl, and gynostemium (fused male and female reproductive organ at central whorl) with diversified shapes and color patterns has captured the vision of people worldwide for hundreds of years [2]. The floral features of orchid, especially the delicate and complex structures of labellum and gynostemium, attract and interact with pollinators to achieve reproductive assurance. In addition to floral diversification, orchids also evolved distinct lifestyles, such as terrestrial growth, epiphytism, and mycoheterotrophy, for adaptation and radiation of heterogeneously ecological niches. It is well established that all orchid species could be monophylletically gathered into Orchidaceae composed of five subfamilies including Apostasioideae (most basal), Epidendroideae, Vanilloideae, Cypripedioideae, and Orchidoideae.

The previous version of OrchidBase 4.0 accommodates the Sanger-sequenced transcriptomes from 11 various tissues of three *Phalaenopsis* species, Illumina-sequenced floral transcriptomes of 10 orchid species across five Orchidaceae subfamilies, as well as whole-genome sequence of *Phalaenopsis equestris* (Epidendroideae) [3], *Dendrobium catenatum* (Epidendroideae) [4], and *Apostasia shenzhenica* (Apostasioideae) [5] and their relative transcriptomes [6–9]. However, the complete genomic sequences from Vanilloideae, Cypripedioideae, and Orchidoideae are still absent in the OrchidBase, hindering the genetic comparisons among orchid species distributed in more than two different orchid subfamilies.

Taxonomic diversity of orchids was obviously observed in two recently expanded sister-subfamilies: Orchidoideae (occupying about 14% species in Orchidaceae) and especially Epidendroideae (about 84%). Floral morphological differences between Orchidoideae and Epidendroideae includes loss of stamen vasculature in Orchidoideae and development of hard pollinia in Epidendroideae [10]. In addition, species in Orchidoideae have a shared terrestrial habitat while those in Epidendroideae are epiphytic growth.

**Fig. 1:**
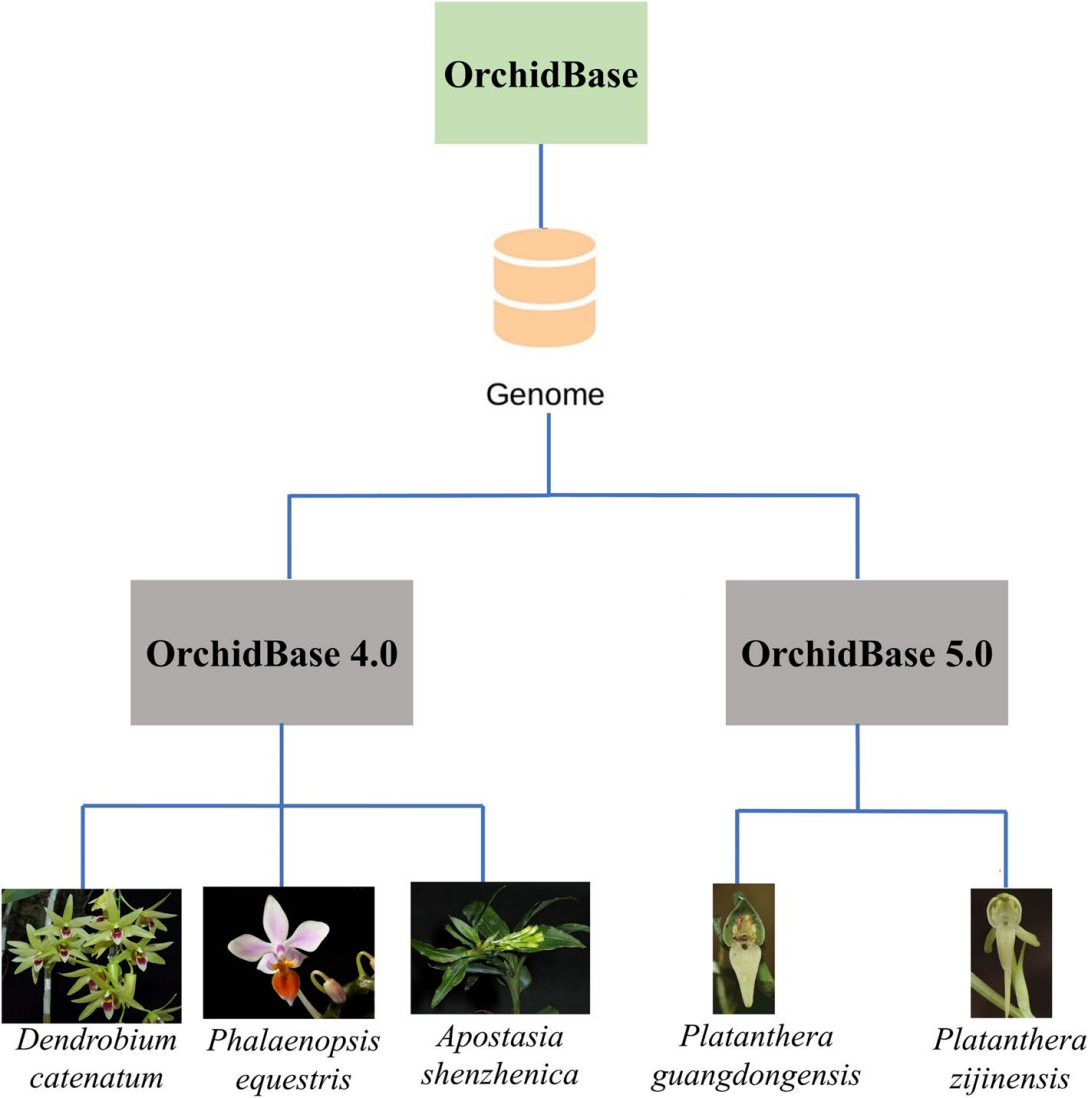
Two newly sequenced orchid genomes (*Pl. zijinensis* and *Pl. guangdongensis*) are added in OrhcidBase 5.0. In total, OrchidBase 5.0 contains the genome information of five orchid species. The pictures of five orchid species were taken by the authors

Recent whole-genome sequenced orchids just concentrated on the species in Epidendroideae, Vanilloideae, and Apostasioideae [3–5, 11–16]. Thus, increasing the genome information of species in Orchidoideae as well as in Cypripedioideae is very important for understanding the orchid genome evolution and the gene function. In the OrchidBase 5.0, we added two whole-genome sequenced Orchidoideae species, *Platanthera zijinensis* and *Platanthera guangdongensis* (Fig. 1), and their transcriptomes derived from various tissues. Furthermore, three tools were newly developed for synteny, gene order and miRNA comparison among five orchid genomes. The data and tools launched in the OrchidBased 5.0 make it an excellent resource for orchid biology research.

## Construction and content

### Implementation and architecture

**Fig. 2:**
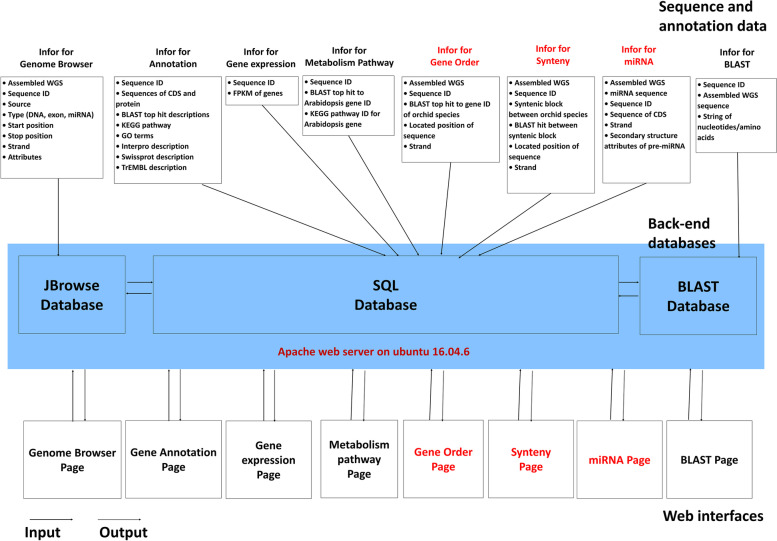
Overview of OrchidBase 5.0 architecture. Three web pages (the synteny page, gene order page and miRNA page) are newly developed in OrchidBase 5.0

The architecture of OrchidBase 5.0 is composed of a MySQL database, a Django application, and a web interface. MySQL 5.7 database is used to store the collected orchid genome sequences and the annotation data. The Django 1.11.1 application executes all the analysis tasks. The python scripts were used to process the orchid genome sequences and annotation data. The web interface is constructed using HTML and Javascript. All the tables were produced by DataTables (a plug-in for the jQuery JavaScript library). All the interactive plots of synteny and gene order were generated by D3.js (a feature-rich JavaScript library). OrchidBase 5.0 is deployed in a workstation with the Linux operation system Ubuntu 16.04.6. OrchidBase 5.0 adopted JBrowse to navigate orchid genomes for visualizing diverse types of genome information. Figure 2 shows the overview of the database architecture. In addition, the content of the database (data and tools) is summarized in Table 1. The Genome Browser, MySQL and BLAST database (Fig. 2) are implemented in the hardware of a workstation with two CPUs, 55 GB RAM and 3.6 TB hard disk space.

**Table 1:** Summary of data and tools that could be browsed and used for the five orchid species (*Phalaenopsis equestris*, *Dendrobium catenatum*, *Apostasia shenzhenica, Platanthera zijinensis*, and *Platanthera guangdongensis*)

**Genome browser**	Scaffold ID, Scaffold sequence, Gene model, miRNA
**Gene annotation**	Gene ID, Gene sequence, BLAST top hit descriptions, KEGG pathway, GO terms, Interpro description, Swissprot description, TrEMBL description, miRNA
**Gene expression**	Gene ID, FPKM values in various tissues
**Metabolism pathway**	Gene ID, Genes mapped to the KEGG pathways
**Gene order**	Gene ID, Physical positions of genes
**Synteny**	Gene ID, Physical positions of genes
**miRNA-targets information**	miRNA gene ID, Structure of miRNA, Target gene IDs of miRNA, Binding sites in the target genes of a miRNA
**BLAST tools**	BLASTN, BLASTX, tBLASTX, BLASTP, tBLASTN

**Fig. 3:**
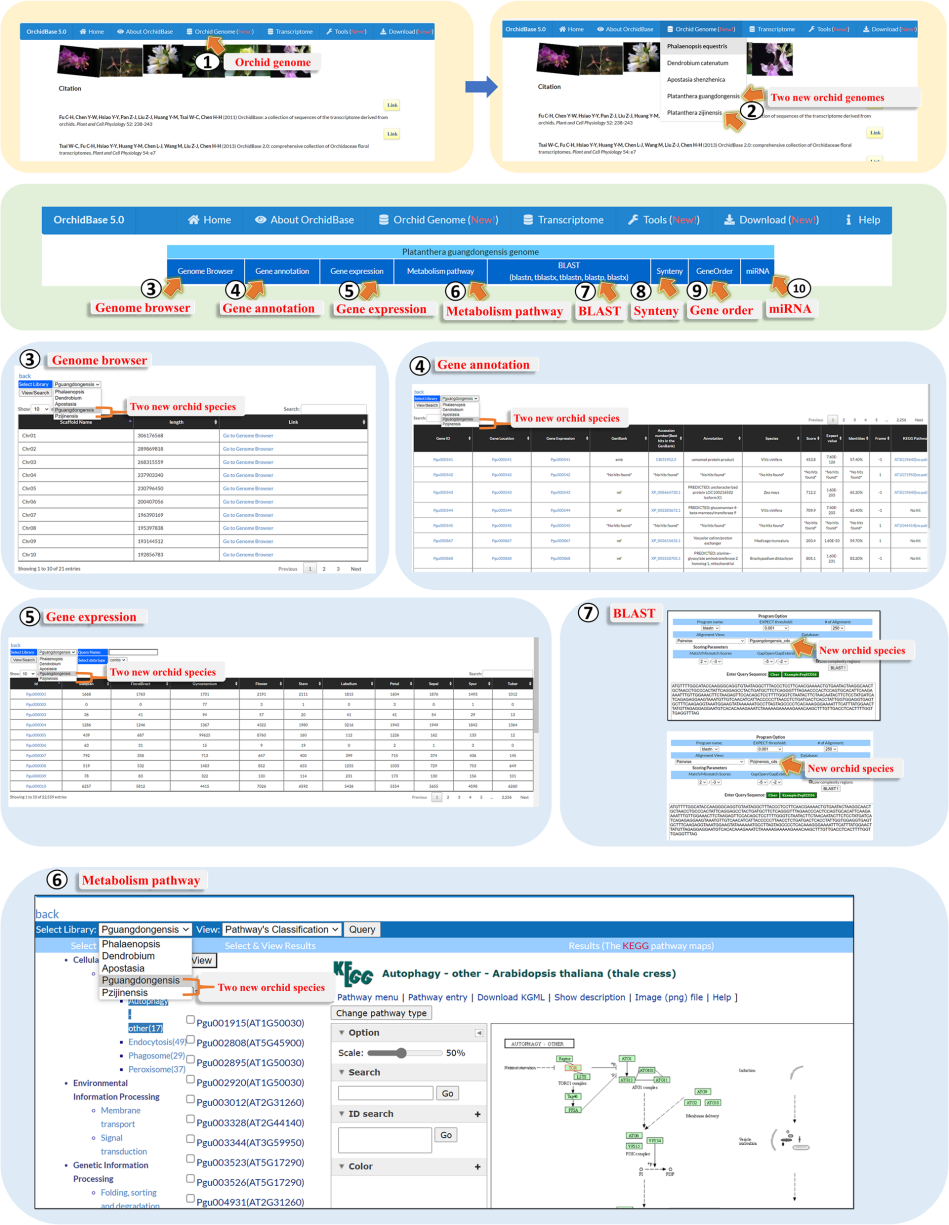
Orchid genome web pages. OrchidBase 5.0 adds the genome and transcriptome information of two newly sequenced orchid species (*Pl. zijinensis* and *Pl. guangdongensis*). The pictures of orchid species were taken by the authors

Figure 3 shows the main page of each orchid species in OrchidBase 5.0 which includes genome (described in OrchidBase 4.0) and transcriptome (described in OrchidBase and OrchidBase 2.0) information. The OrchidBase 5.0 simplifies the workflow for huge and complicated biological data analysis and visualization. OrchidBase 5.0 is an open-access, web-available portal that integrates the available data for the genomes of the five orchid species (*P. equestris*, *D. catenatum*, *A. shenzhenica*, *Pl. zijinensis*, and *Pl. guangdongensis*) and the related transcriptome information.

### Expanded database content

*Pl. zijinensis* and *Pl. guangdongensis* both have a karyotype of 2 N = 2X = 42 chromosomes. A total of 441.16 Gb and 414.20 Gb data were generated respectively for *Pl. zijinensis* and *Pl. guangdongensis* using PacBio technologies [17]. The total length of the genome assembly was 4.19 Gb with a contig N50 value of 1.77 Mb for the *Pl. zijinensis* genome and 4.20 Gb with a contig N50 value of 1.57 Mb for the *Pl. guangdongensis* genome (Table 2) [17]. Hi-C libraries were built and sequenced by Illumina technology to reconstruct physical maps by ordering and clustering the assembled scaffolds into 21 pseudomolecules for each species. The raw data and whole genome-assembled scaffold sequences of the two *Platanthera* species (BioProject PRJNA739531) were downloaded from the National Center for Biotechnology Information (NCBI) database. Statistics of the added orchid genomes is presented in Table 2. Based on these datasets, protein-coding genes were predicted by combination of homology-based prediction, de novo gene prediction, RNA sequence-aided prediction [18] for each species. Twenty-four thousands five hundreds and thirteen and 22,559 protein-coding genes in the respective genome of *Pl. zijinensis* and *Pl. guangdongensis* were predicted. Furthermore, 31 and 33 miRNAs were identified in the *Pl. zijinensis* and *Pl. guangdongensis* genomes, respectively [17]. Each predicted gene is assigned to a specific Gene ID as the identifier of the predicted gene. The specific genes could be selected to investigate their annotated functions involved in biological processes of interest.

**Table 2:** Comparison of the assembled genomes among the five orchid species collected in the OrchidBase 5.0

Orchid species	Assembled genome size	N50 length of scaffold	N50 length of contig	Number of predicted genes	Reference
*Phalaenopsis equestris*	1.13 Gb	1.22 Mb	45.8 Kb	29,545	Zhang et al*.*, 2017 [5]
*Dendrobium catenatum*	1.12 Gb	1.06 Mb	51.7 Kb	29,257	Zhang et al*.*, 2017 [5]
*Apstasia shenzhenica*	349 Mb	3.03 Mb	80.1 Kb	21,841	Zhang et al*.*, 2017 [5]
*Platanthera zijinensis*	4.19 Gb	Not determined	1.77 Mb	24,513	Li et al*.*, 2022 [17]
*Platanthera guangdongensis*	4.20 Gb	Not determined	1.57 Mb	22,559	Li et al*.*, 2022 [17]

The transcriptomics data derived from the two *Platanthera* species were also downloaded from BioProject PRJNA739531. All RNA sequencing (RNA-seq) reads were mapped to the predicted genes and the fragments per kilobase of transcript per million mapped reads (FPKM) values for each gene in various tissues and different developmental stages were calculated. All the gene expression values have been integrated into the updated version of OrchidBase 5.0.

## Utility and discussion

### Searching the genome information of the two Orchidoideae species in the database

Through the web interface, the genome information of the two *Platanthera* species contained in OrchidBase 5.0 could be freely obtained. The information can be linked via the “Orchid Genome" icon (Fig. 3, step 1). With the interface, a page allows users to select one of the three already existed orchid genomes (*P. equestris*, *D. catenatum*, *A. shenzhenica*) or two newly added orchid genomes (*Pl. zijinensis*, and *Pl. guangdongensis*) (Fig. 3, step 2). Users then could access the Genome browser (Fig. 3, step 3), Gene annotation (Fig. 3, step 4), Gene expression (Fig. 3, step 5), Metabolism pathway (Fig. 3, step 6), BLAST (Fig. 3, step 7), Synteny (Fig. 3, step 8), Gene order (Fig. 3, step 9), and miRNA (Fig. 3, step 10) for querying the genome and obtain the gene information and comparison analysis in the selected orchid genome. Owing to Genome browser, Gene annotation, Gene expression, Metabolism pathway, and BLAST have been introduced in OrchidBase 4.0 [9], here we only explained the new pages of Synteny, Gene order and miRNA in details.

### Database user protocol

#### “Synteny” page

**Fig. 4:**
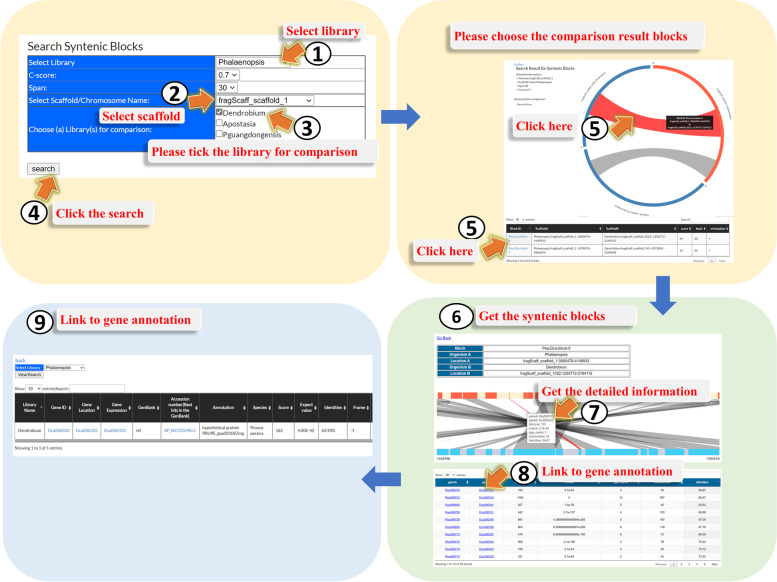
A step by step guide for the “Synteny” page. The detailed descriptions could be seen in “Database user protocol” section

Synteny is a locally conserved gene order found in the compared genomes. Two species that have recently diverged from a common ancestor might be expected to share a similar set of genes positioned along the DNA strand in the same order. Genomic synteny comparisons among species have provided an additional opportunity to study evolutionary trajectories that lead to diversity of chromosomal structure in many lineages [19]. The “Synteny” page is a graphical interface for displaying syntenic relationships (predicted using MCScanX [20]) between the selected genomic region and potential orthologous region of the other genome (Fig. 4). For visiting the “Synteny” page, users could click the Orchid Genome (Fig. 3, step 1), and then choose one of the orchid species (Fig. 3, step 2). Users will be directed to the main page of the selected genome and then enter the “Synteny page” (Fig. 3, step 8). In the “Synteny page”, users could select again one of the orchid species (Fig. 4, step 1). Users then could anchor one of the scaffolds (Fig. 4, step 2) in the selected orchid species and tick the other orchid genome for further comparison (Fig. 4, step 3). When users click the “search” icon at the left corner of the page (Fig. 4, step 4), they will be lead to the next page for showing the result indicating the orthologous blocks between the selected genomic scaffolds. In the page, users could pick one of the syntenic blocks (Fig. 4, step 5) to further understand the relative position of the genes in the syntenic block (Fig. 4, step 6) and get the detailed gene information (Fig. 4, step 7 and 8) and also gene annotation linked to the Gene Annotation page (Fig. 4, step 9).

#### “Gene Order” page

**Fig. 5:**
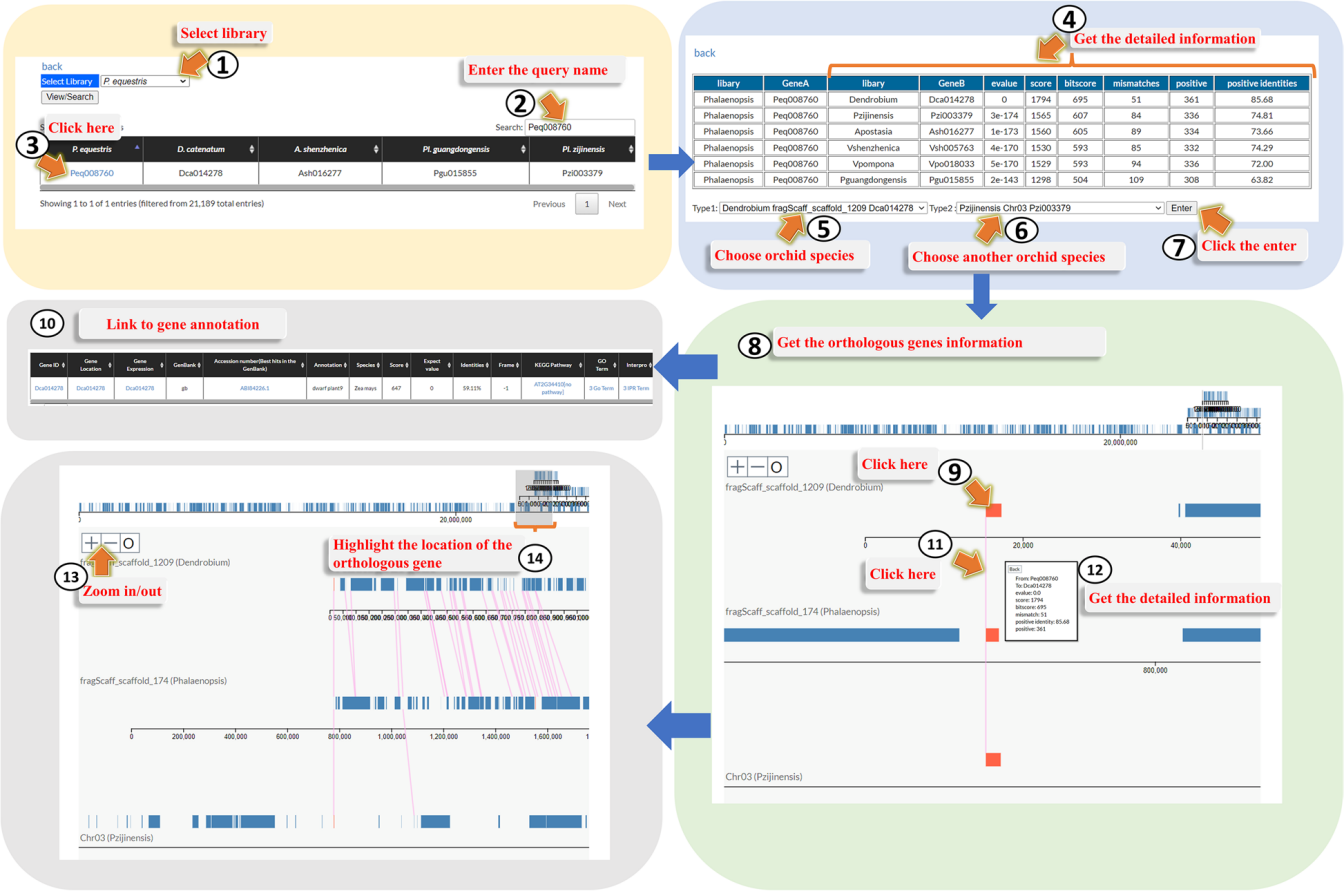
A step by step guide for the “Gene order” page. The detailed descriptions could be seen in “Database user protocol” section

We provided another way to display permutation of gene arrangement based on the BLASTP and genomic location of genes, and the system we named here is “Gene Order” (Fig. 5). Users would not necessary to specify a scaffold, but select a unique “gene ID” of the genome. Based on the best hit of the BLASTP, the system provides the location of the orthologous genes in the other two selected genomes, and further indicates the position of the other homologous genes among the three compared species. To help users execute “Gene Order” analysis, an intuitive graphic interface was developed (Fig. 5). By accessing the “Gene Order” page, orchid genome could be selected (Fig. 3, step 1), and one of the orchid species could be opted (Fig. 3, step 2). In the main page of the selected orchid species, users could click the Gene order (Fig. 3, step 9). In the “Gene Order” page, users have to select one of the orchid species (Fig. 5, step 1) and offer the Gene ID (Fig. 5, step 2) for showing the best BLASTP hits among the other orchid genomes. The Gene ID marked by blue color could be clicked (Fig. 5, step 3), then users would be directed to the next page presenting the detailed comparison results among orchid orthologous proteins (Fig. 5, step 4). In this page, users could further select two orchid genomes (Fig. 5, step 5 and 6). After clicking the “Enter” icon (Fig. 5, step 7), the comparison of gene order among the three chosen genomes will be displayed (Fig. 5, step 8). In this page, the red rectangle at the middle row is the specified gene in the orchid genome selected in (Fig. 5, step 1), the red one at the top row is the orthologous gene in the genome selected in (Fig. 5, step 5), and the red one at the bottom row is another orthologous hit in the genome selected in (Fig. 5, step 6). User could point each of the colored rectangle to have the annotated information of the gene (Fig. 5, step 9 and 10) or each line connected between two orthologs to get the detailed comparison information (Fig. 5, step 11 and 12). The page also provides “Zoom in” and “Zoom out” function (Fig. 5, step 13) to enlarge or narrow specific region in the scaffold of the selected genome and offers a movable gray region to navigate the selected zone (Fig. 5, step 14).

#### “miRNA” page

**Fig. 6:**
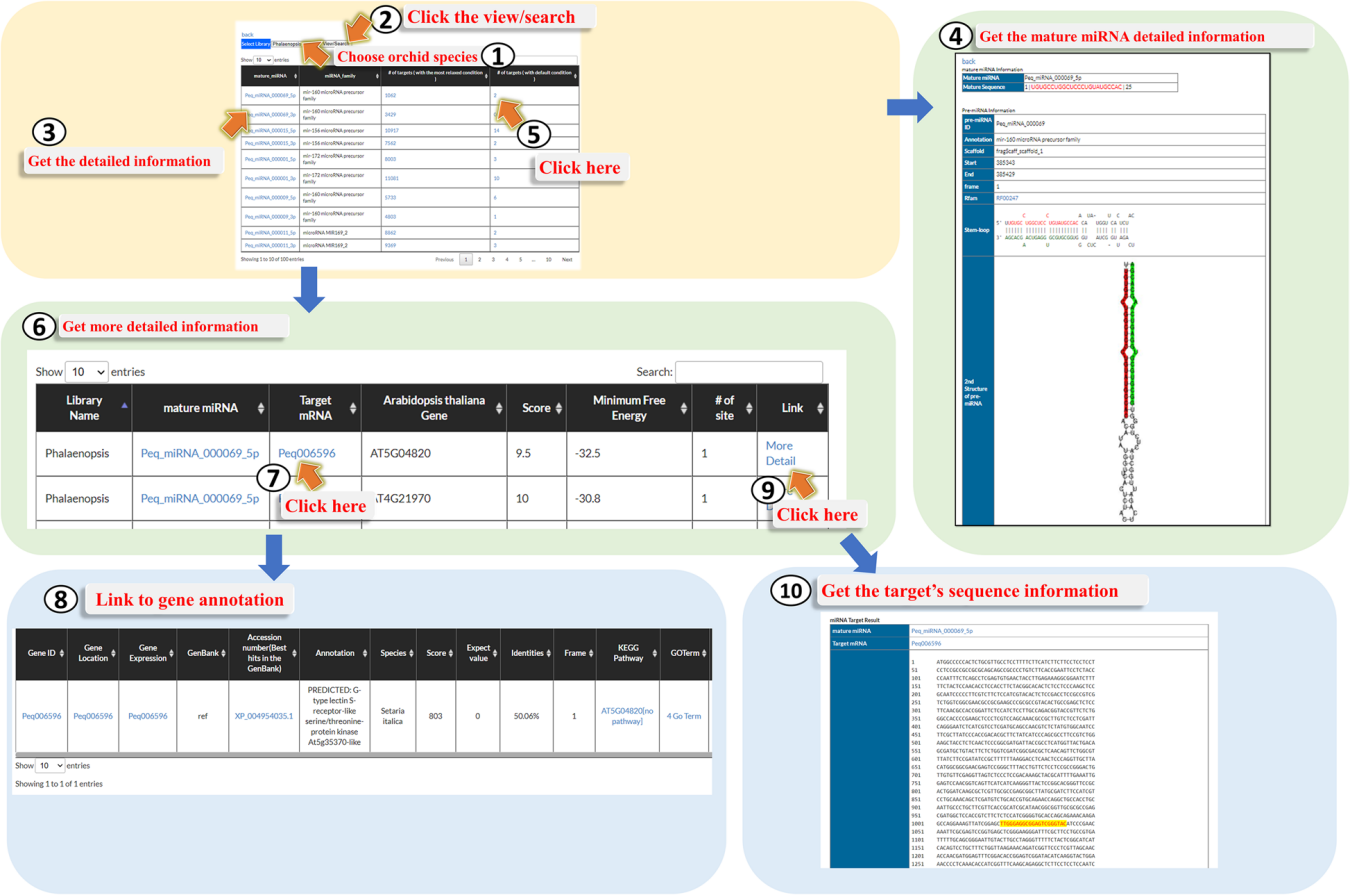
A step by step guide for the “miRNA” page. The detailed descriptions could be seen in “Database user protocol” section

miRNAs are small noncoding RNAs involved in regulating many biological processes at the post-transcriptional and translational levels. The molecular function of miRNAs is suppression the expression of their targets. For providing pre-miRNAs of orchid genomes, we identified them by using INFERNAL [21] to search the Rfam database. We further used miRLocator [22] to predict mature miRNAs. For identification of orchid miRNA targets, we uploaded the mature miRNAs sequences and orchid coding sequences (CDSs) to TAPIR [23], and adopted RNAhybrid search engine equipped in the server to get the target sequences. For going to the miRNA page, users could visit the Orchid Genome page (Fig. 3, step 1), then select one of the orchid species (Fig. 3, step 2). Users will step into the main page of the selected orchid species and then could click the miRNA page (Fig. 3, step 10). In the miRNA page, users have the further choice to pick one of the orchid species (Fig. 6, step 1) and click the View/Search item to browse the miRNAs in the selected orchid species (Fig. 6, step 2). Users also could type “miRNA ID” or keywords to query the miRNA information (Fig. 6, step 3). In this page, users could get more detailed information by accessing one of the “mature miRNAs” to obtain mature miRNA sequence, its annotation, and secondary structure of its pre-miRNA (Fig. 6. step 4). Users could also click one of the “number of targets” to visualize the “target mRNA” information (Fig. 6, step 5), and find the target gene names (Fig. 6, step 6). Users could click the target gene name (Fig. 6, step 7) and go to the “Gene Annotation” page (Fig. 6, step 8). Finally, the miRNA targeting site in the target mRNA could be shown by clicking one of choice at the “Link” (Fig. 6, step 9 and step 10).

## A case study

**Fig. 7:**
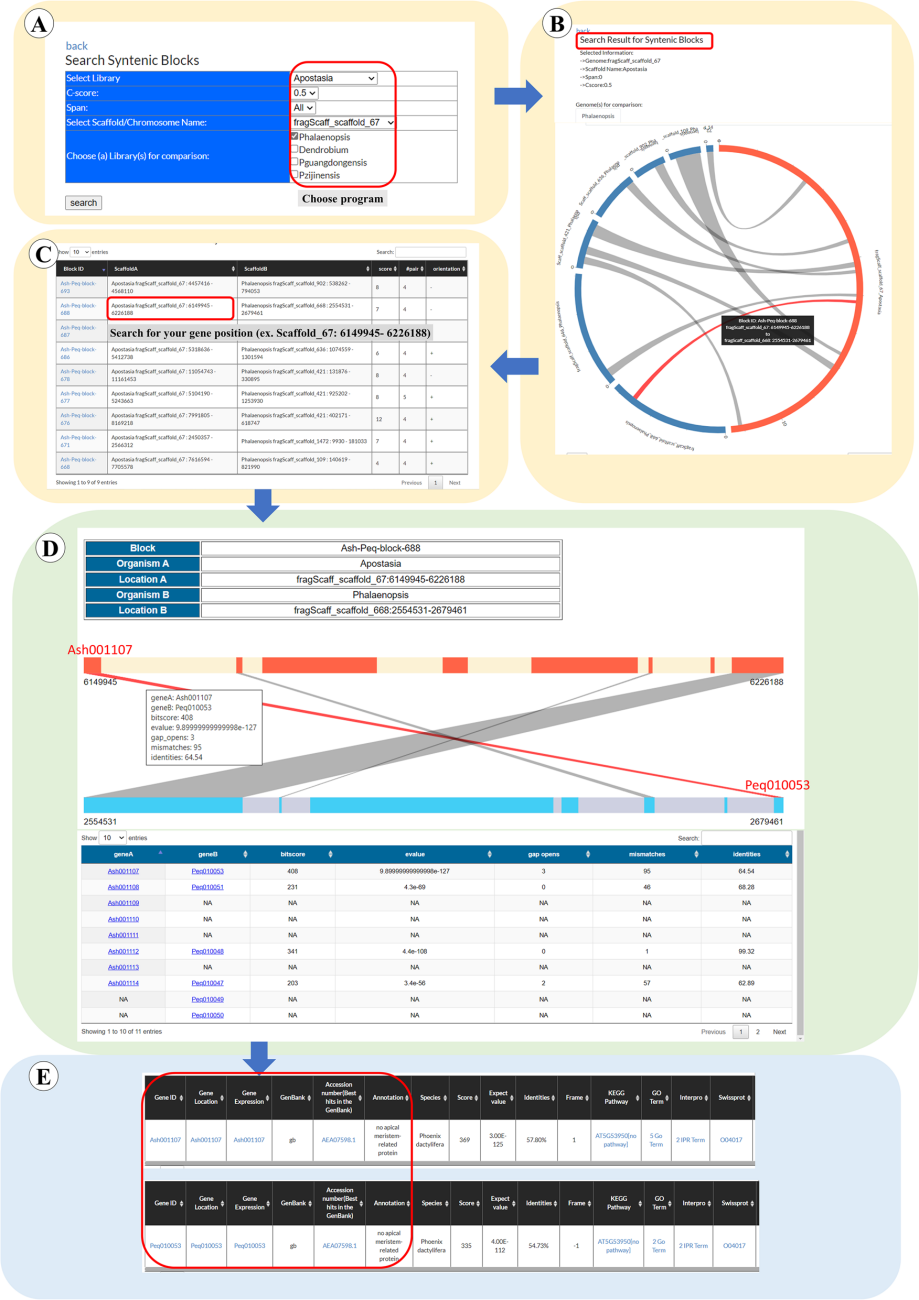
An example displaying “Synteny” analysis of *Apostasia CUC*-like gene *Ash001107* and *Phalaenopsis CUC*-like gene *Peq010053*. **A** We selected the library as “*Apostasia*”, Scaffold/Chromosome as “fragScaff_scaffold_67”, and chose any one of the orchid genomes (in this case: *Phalaenopsis*), then clicked search. **B** The page was changed to the synteny blocks screened between *Apostasia* and *Phalaenopsis* genomes. **C** We chose one of the lines connected *Apostasia* and *Phalaenopsis* blocks and searched the gene location to match *Phalaenopsis* scaffold harboring *CUC* gene. **D** We selected the synteny block containing *Apostasia Ash001107* to show the detailed information of this region embedding order and direction of genes and orthologous relationship among each gene. **E** We found that the *CUC* gene in *Phalaenopsis* is *Peq010053* and the *CUC* genes in these two orchid species formed a synteny block. Finally, the *Ash001107* and *Peq010053* could be clicked to reveal their gene annotation information

*NO APICAL MERISTEM* (*NAC*), *ARABIDOPSIS THALIANA ACTIVATING FACTOR* (*AtAF*), and *CUP-SHAPED COTYLEDON* (*CUC*) encode distinct members of the plant *NAC* family [24]. This family is suggested to be specific for land plants [25]. Previous researches concluded that *CUC*s have conserved function in gynoecium and ovule development across angiosperms and some of *CUC* transcripts (for example *Arabidopsis CUC1* and *CUC2*) were regulated by *miR-164* [26]. Because the orchid gynostemium (fused by androecium and genoecium floral organs) is distinct from those in most angiosperm species, we are interested in the function of *CUC* homologs played in orchid gynostemium development. *Arabidopsis CUC1* (NCBI accession number: BAB20598) was used as query to BLASTP against *Apostasia* protein database, and the *Apostasia Ash001107* located at fragScaff_scaffold_67: 6,149,945–6,151,832 was hit. We then went to “Synteny” page (Fig. 7) and selected library as “*Apostasia*”, Scaffold/Chromosome as “fragScaff_scaffold_67”, and chose any one of the orchid genomes (in this case: *Phalaenopsis*), then clicked search (Fig. 7A). The page was changed to the synteny blocks screened between *Apostasia* and *Phalaenopsis* genomes (Fig. 7B). We chose one of the lines connected *Apostasia* and *Phalaenopsis* blocks and searched the gene location to match *Phalaenopsis* scaffold harboring *CUC* gene (Fig. 7C). We selected the synteny block containing *Apostasia Ash001107* to show the detailed information of this region embedding order and direction of genes and orthologous relationship among each gene (Fig. 7D). We found that the *CUC* gene in *Phalaenopsis* is *Peq010053* and the *CUC* genes in these two orchid species formed a synteny block. Finally, the *Ash001107* and *Peq010053* could be clicked to reveal their gene annotation information (Fig. 7E).

**Fig. 8:**
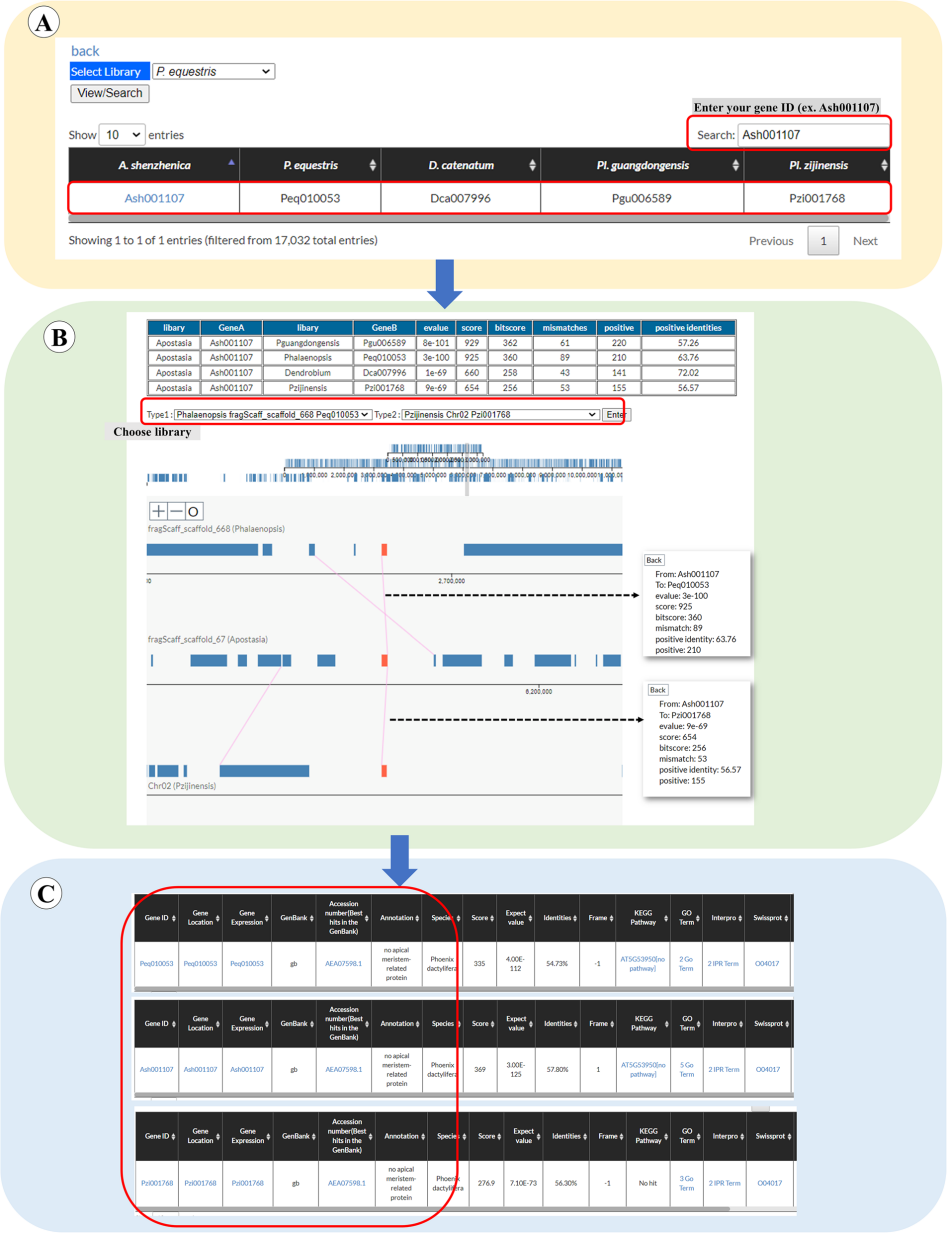
An example displaying “Gene Order” analysis of *Apostasia CUC*-like gene *Ash001107* among *Apostasia*, *Phalaenopsis*, and *Pl. guangdongensis*. **A** We filled in the gene ID (in this case: *Ash001107*) into the “Search” blank at the “Gene Order” page and the putative orthologs in other orchid genomes were shown. **B** The *Ash001107* marked by blue color was clicked. We then selected two orchid genomes for comparison (in this case: *Phalaenopsis* and *Pl. guangdongensis*). **C** We also could click the red or blue rectangle to reveal the specific gene annotation

“Gene Order” also could be applied to reveal the permutation of gene arrangement among orchid genomes. We filled in the gene ID (in this case: *Ash001107*) into the “Search” blank at the “Gene Order” page and the putative orthologs in other orchid genomes were shown (Fig. 8A). The *Ash001107* marked by blue color was clicked. We then selected two orchid genomes for comparison (in this case: *Phalaenopsis* and *Pl. guangdongensis*, Fig. 8B), and clicked “Enter”. We clicked “ ± ” to zoom in/out the region around the indicated gene ID (marked by red color) to adjust the comparison region among the selected orchid genomes (Fig. 8B). We found that the regions around the *CUC* genes in *Apostasia* and *Phalaenopsis* may have synteny relationship since many genes in the compared regions are orthologs. On the contrary, the regions around the *CUC* genes in *Apostasia* and *Pl. guangdongensis* may not have synteny relationship since many neighboring genes of the *CUC* gene in *Apostasia* could not find orthologs nearby the *CUC* gene in *Pl. guangdongensis*. We also could click the red or blue rectangle to reveal the specific gene annotation (Fig. 8C). One of the advantages of “Gene order” tool compared to the “Synteny” is that if the putative orthologs do not locate in the corresponding synteny block, the putative orthologs and its neighboring genes could still be displayed and compared. The other advantage is that the “Gene order” tool could simultaneously compare three orchid genomes.

**Fig. 9:**
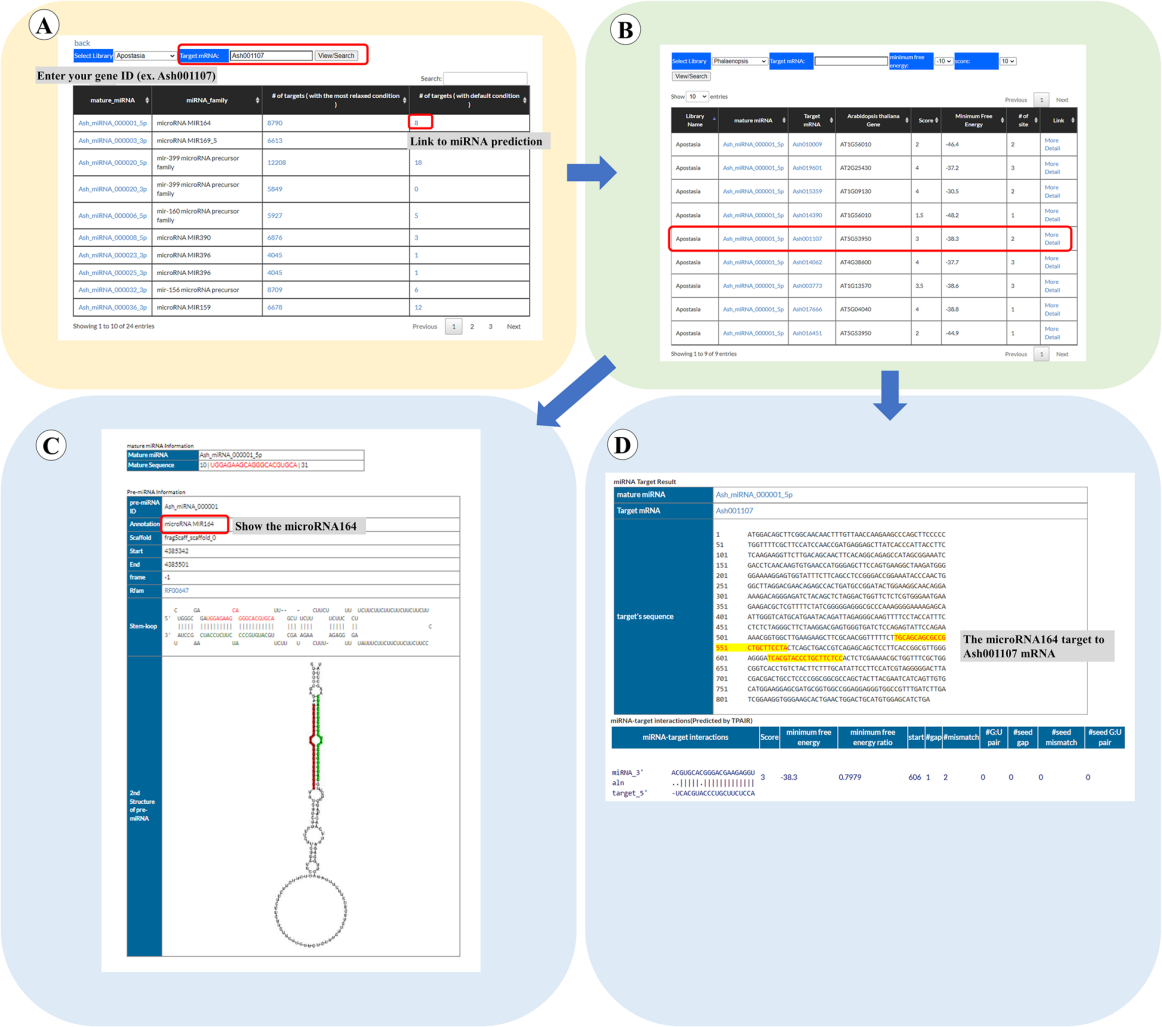
An example displaying “miRNA” analysis of *Apostasia CUC*-like gene *Ash001107* which is regulated by *miR-164*. **A** We entered “miRNA” page, and filled *Ash001107* in the “Target mRNA”, then clicked “View/Search”. The results showed that *Ash001107* is regulated by 24 different miRNAs, one of which is *Ash_miRNA_000001_5p*. **B** *Ash_miRNA_000001_5p* is the *miR-164* in *Apostasia* and it has eight targets. **C** The detailed information of *Ash_miRNA_000001_5p* is shown. **D** The detailed information of the *miR-164* binding sites in *Ash001107* mRNA is shown

*Arabidopsis CUC* genes were known to be regulated by *miR-164* [26]. To check whether *Ash001107* (*CUC* gene in *Apostasia*) is also regulated by *miR-164*, we entered “miRNA” page, and filled *Ash001107* in the “Target mRNA”, then clicked “View/Search”. The results showed that *Ash001107* is regulated by 24 different miRNAs, one of which is *Ash_miRNA_000001_5p* (Fig. 9A). Note that *Ash_miRNA_000001_5p* is the *miR-164* in *Apostasia* and it has eight targets (Fig. 9B and C). Therefore, we concluded that *CUC* gene in *Apostasia* is also regulated by *miR-164*. The detailed information of the *miR-164* binding sites in *Ash001107* mRNA could be seen in Fig. 9D. The miRNA binding site information is useful for biologists to design experiments for exploring the regulatory mechanism of *CUC*-like gene controlled by *miR-164* in orchid.

## Conclusions

We added two whole genome sequences of Orchidoideae species (*Pl. zijinensis* and *Pl. guangdongensis*) and their transcriptomes into OrchidBase 5.0. In addition, two functions for genome comparison (synteny and gene order) and miRNA characterization were developed in this version. Both expanding the Orchidoideae genomes and creating new tools for exploring knowledge embedded in the nucleotide sequences provide the opportunity for the users to get novel insights into the conservation and diversification of orchid genomes. Users could precisely and efficiently adopt “synteny” and “gene order” tools to find specific collinear region among the five orchid genomes (*P. equestris*, *D. catenatum*, *A. shenzhenica*, *Pl. zijinensis* and *Pl. guangdongensis*). Although studies of miRNA function involved in orchid growth and development are still scarce [27, 28], we believe that the researches will be increased after the miRNA characterization tool in the OrchidBase 5.0. has been released. In the future, we will continue to include new orchid whole genome sequences in the OrchidBase and also develop new -omic analysis tools for plant scientists.

## Data Availability

The raw data and whole genome-assembled scaffold sequences for the two *Platanthera* species (BioProject PRJNA739531) were downloaded from the National Center for Biotechnology Information (NCBI) database. The transcriptome data derived from the two *Platanthera* species were also downloaded from BioProject PRJNA739531 (https://www.ncbi.nlm.nih.gov/bioproject/?term=PRJNA739531).

## Abbreviations

*A.**shenzhenica*: *Apstasia* *shenzhenica*
*D. catenatum*: *Dendrobium catenatum*
KEGG: Kyoto Encyclopedia of Genes and Genomes
FPKM: Fragments Per Kilobase of transcript per Millions mapped reads
GO: Gene Ontology
*P.**equestris*: *Phalaenopsis* *equestris*
*Pl.**zijinensis*: *Platanthera* *zijinensis*
*Pl.**guangdongensis*: *Platanthera* *zijinensis*
SQL: Structured Query Language
